# Editorial: Modulators of Cross-Language Influences in Learning and Processing

**DOI:** 10.3389/fpsyg.2022.898793

**Published:** 2022-05-16

**Authors:** Tamar Degani, Anat Prior, Zofia Wodniecka

**Affiliations:** ^1^Department of Communication Sciences and Disorders, University of Haifa, Haifa, Israel; ^2^Department of Learning Disabilities and Edmond J. Safra Brain Research Center for Learning Disabilities, University of Haifa, Haifa, Israel; ^3^Psychology of Language and Bilingualism Lab, Institute of Psychology, Jagiellonian University, Krakow, Poland

**Keywords:** bilingual, cross-language influences, transfer, second language learning, multilingualism, lexicon, morphosyntax

## Introduction

Language learning and processing should be considered in the context of speakers' prior linguistic knowledge. Research accumulated over the last few decades (Jarvis and Pavlenko, 2007) has indeed demonstrated that *cross-language influences* (CLI), also termed *transfer*, are present across different language domains, for bi- and multilinguals (Cenoz et al., 2001; Puig-Mayenco et al., 2020). Research on CLI is important for several reasons. On the theoretical front, such evidence can reveal the patterns of interconnectivity of the multilingual language system and inform models of multilingual representation and activation. Further, such research carries implications for learning and instruction, in understanding when and how CLI from prior linguistic knowledge would facilitate or hinder learning.

**Figure 1:**
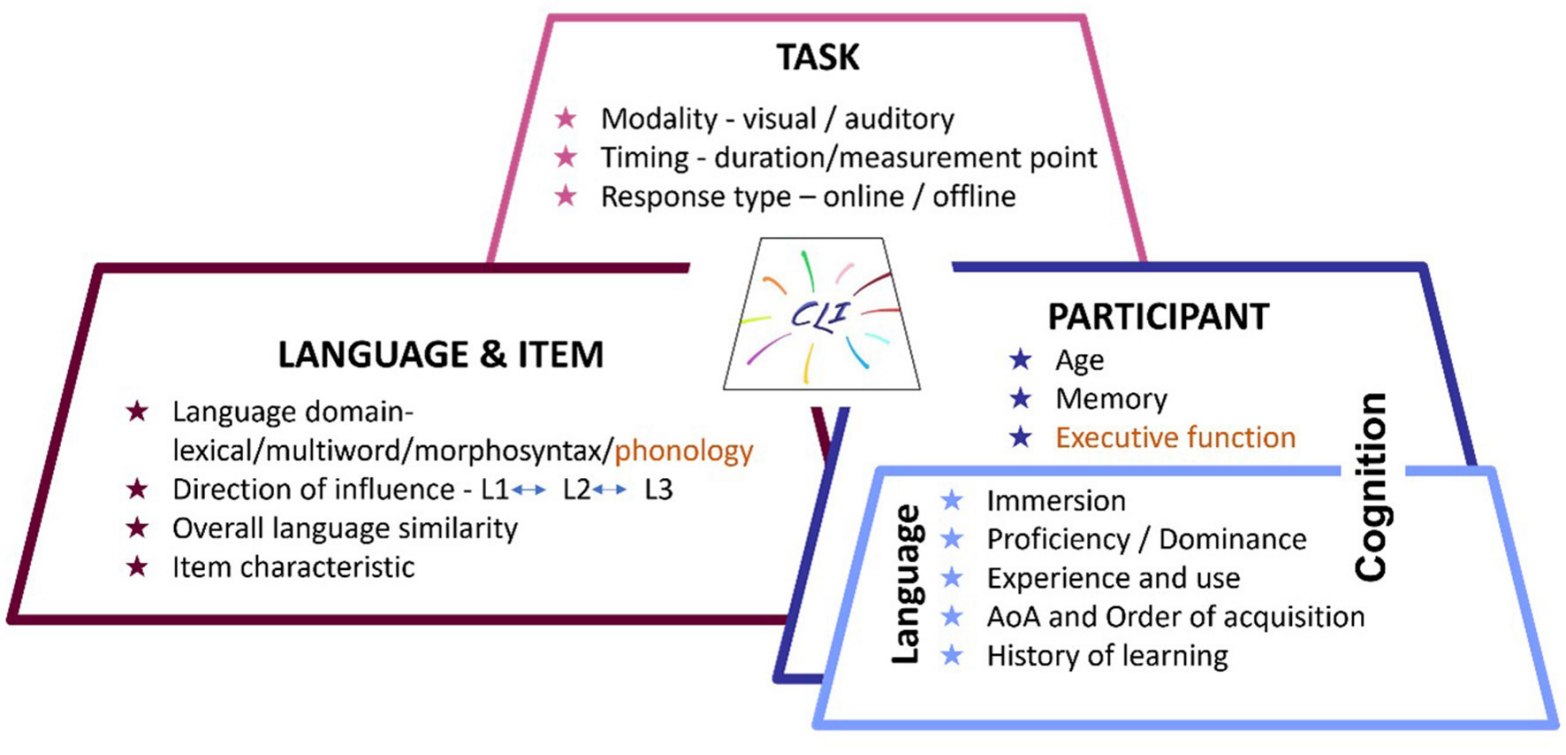
Modulators of CLI. Figure presents modulators of CLI identified in the current volume grouped by category. In orange, we also include modulators not represented in the current volume but which we believe might be important for future research.

Despite wide agreement regarding the prevalence and importance of CLI, there is quite a lot of variability in its specific manifestations across studies. Thus, the goal of the current Research Topic is to set the stage for systematically mapping the factors that may modulate the presence and nature of CLI in learning and processing. Studies included in this Research Topic investigate CLI in children and adults, across lexicon and grammar, in beginning and advanced language users. Importantly, the studies identified and tested factors that might modulate CLI. Across the papers, the modulators examined include speaker characteristics, task demands, and item/language characteristics (see Figure 1), thus offering a rich and nuanced description of the factors at play. In what follows, we present the collection of studies in this Research Topic according to the language domain on which they focused (see Table 1), as well as outline commonalities and avenues for future research.

**Table 1:** Overview of studies in the Research Topic.

**Authors**	**CLI assessment**	**Experimental paradigm**	**Population**	**CLI facilitation/ interference**	^*^Modulators tested (observed)	**Direction tested (observed)**
**Lexical domain**						
Fricke	Cognate status	Auditory lexical decision	▪ English monolingual adults ▪English-Spanish learners (late) ▪Spanish-English (heritage)	Cognate facilitation	▪Type of background noise (no) ▪Variation in linguistic experience (yes) ▪L1 and L2 proficiency (yes)	L1 on L2 (yes)
Hoshino et al.	Semantic, phonological, or translation word overlap	Picture naming in picture-word interference	▪Spanish-English adults in US ▪Japanese-English adults in US	In both groups: ▪Translation facilitation Only in Spanish-English group: ▪Semantic interference Phonological facilitation Phono-translation facilitation	▪Script (yes) ▪Type of cross-language similarity (yes)	L1 on L2 (yes)
Iniesta et al.	▪Cognate status ▪Degree of orthographic and phonological similarity	Writing to dictation	English-Spanish adults in US	▪Facilitation ▪Interference	▪Speakers' language background: heritage vs. late learners (yes) ▪Type of cross-linguistic similarity (yes)	L1 on L2 (yes) L2 on L1 (yes)
Lipner et al.	Lexical depth and breadth as a function of language intervention	Vocabulary intervention	English-Hebrew children in IL	Semantic facilitation (knowledge transfer, qualitatively observed)	▪Vocabulary dominance at baseline (yes) ▪Language proficiency (yes) ▪Age of acquisition (yes) ▪ Type of language: heritage vs. second (yes)	L1 on L2 (yes) L2 on L1 (yes)
Marian et al.	Cross-language word similarity	Self-paced paired-associate learning task	English monolingual adults in US	▪Facilitation due to similarity at early stages of learning ▪Interference due to similarity at later stages	▪Similarity with native language words (yes) ▪Sequence of learning (yes)	L1 on L2 (yes)
Whitford and Joanisse	Within- and cross-language word form similarity	Naturalistic paragraph reading, eye-tracking	French-English adults and children in Canada	Facilitation for similar word forms	▪Age/reading experience (yes) ▪Orthographic neighborhood density (yes)	L1 to L2 (yes) L2 to L1 (yes)
Woumans et al.	Cognate status	Written word production (in response to a picture)	Dutch-English adults in Netherlands	Cognate facilitation	Type of sentence constraint (yes)	L1 on L2 (yes)
**Multi-word expressions**						
Du et al.	Binomials (congruent/incongruent and translated)	Primed visual lexical decision	▪Chinese-English adults in New Zealand ▪English-Chinese adults in China ▪English monolinguals in New Zealand	▪Facilitation for congruent collocations ▪No effect for translated collocations	Language direction (yes)	L1 on L2 (yes) L2 on L1 (yes)
Otwinowska et al.	L1 collocational calques from L2	▪Acceptability judgments ▪Sentence reading, ERPs	Polish-English adults in Poland	▪Facilitation for collocational calques in sentence reading and ERP ▪No effect in acceptability judgments	▪Type of task (yes) ▪Language proficiency (no)	L2 on L1 (yes)
Pulido	L2 collocations (congruent/incongruent with the L1)	Sentence reading, eye-tracking	English (L1) advanced learners of Spanish (L2) in US	Facilitation for congruent collocations	▪L2 Chunking ability (yes) ▪Early vs. late reading measures (no)	L1 on L2 (yes)
**Morpho-syntactic domain**						
Abbas et al.	L3 Grammatical structures non-overlapping with L1, L2 or both	▪English sentence reading, eye tracking; ▪Grammaticality judgements	Arabic-Hebrew-English trilinguals in Israel	Interference from L1 and from L2 in L3 processing. No interference when L1 and L2 overlap with each other and differ from L3	▪Language status (yes) ▪Task/measure (yes)	L1 on L3 (yes) L2 on L3 (yes)
Kubota et al.	▪Genitive structures (partially overlapping) ▪Verb argument structure (non-overlapping)	Preference / Grammaticality Judgment	▪Japanese-English bilingual children in Japan, immediately after English immersion, and one year later. ▪English monolingual children in the UK	▪Differences in processing genitive structures (influence by L1) ▪No effect in processing Verb-Argument structures	▪Proficiency and immersion (yes) ▪Grammatical Structure (yes)	L1 on L2 (yes)
Leon Guerrero et al.	Linguistic and meta-linguistic skills in L1 and L2	▪English text reading, eye tracking ▪Comprehension questions	Spanish-English and monolingual English adolescents in the US	Syntactic integration skill in L1 associated with improved L2 reading and comprehension	Text syntactic difficulty (yes)	L1 on L2 (yes)
Meir and Janssen	▪Genitive and ▪Accusative structures in Heritage Language (which do or do not overlap with Societal language)	Russian oral elicitation task	▪Russian-Hebrew bilingual children in Israel ▪Russian-Dutch bilingual children in the Netherlands ▪Monolingual Russian children in Russia	Societal language facilitates acquisition of shared Heritage language structures	▪Language similarity (yes) ▪Participants' proficiency in the Heritage Language (yes)	Societal Language on Heritage Language (yes)
Russak and Zaretsky	Linguistic and meta-linguistic skills in L1, L2, and L3	English oral narrative production	▪Hebrew-English bilingual ▪Arabic-Hebrew-English trilingual children in Israel	Meta-linguistic awareness in Hebrew (L1/L2) associated with improved narrative production in English (L2/L3)	Language group profile (yes)	L1/L2 on L3 (yes)
Vingron et al.	▪Noun adjective order (partially overlapping) ▪Object-pronouns structure (non-overlapping)	English sentence reading, eye tracking	▪French-English bilingual adults ▪English-French bilingual adults ▪English monolinguals In Canada	Faster processing for English violations consistent with French.	▪Grammatical structure (yes) ▪Participants' L2 background and exposure (yes)	L1 on L2 (yes) L2 on L1 (yes)

^*^Some of the effects observed were weak, but we opted to make a binary decision regarding whether modulations and influences were observed. See original papers for more details.

### Lexical Domain

Seven studies in this volume examined the way in which CLI is manifested in learning and processing of single words (Fricke; Hoshino et al.; Iniesta et al.; Lipner et al.; Marian et al.; Whitford and Joanisse; Woumans et al.). Even a quick overview of the studies reveals a rich heterogeneity of the methodological approaches; from eye tracking while reading, through oral, or written production, auditory word recognition, pair-associate learning, to a vocabulary intervention. Across these studies, several factors emerged as modulating CLI, including those that relate to the experimental task, to individual differences across participants in background language and cognition, as well as to item and language specific characteristics. CLI was observed in all the studies, and whenever both directions of influence were tested, bidirectional effects were reported, although the specific manifestation appears to depend on a range of factors, including duration/measurement point and stage of processing.

### Multi-Word Expressions

Going beyond the single word level, three studies in this volume examined the way in which CLI is modulated in the case of multi word units, which are fixed sequences of words that tend to co-occur within a given language. Of interest, the key question guiding the work presented here was whether knowledge of MWE transfers across languages, and what factors modulate such CLI (Otwinowska et al.; Du et al.; Pullido). Here too, a variety of methodologies have been implemented; priming of binomials (e.g., knife and fork, Du et al.), eye tracking while reading collocations, and behavioral and ERP measures of collocation processing. Across these studies, there is evidence for CLI in both directions of influence. Thus, L2 processing appears to be affected by CLI from the LI. At the same time, L1 processing is also affected by CLI from the L2, even when speakers are immersed in their L1 environment. Of note, such influence from L2 to L1 was weaker and evident in some measures but not others.

Interestingly, when stimuli with overlapping representations across languages are compared to non-overlapping controls, a congruency effect leads to facilitated processing (Du et al.; Pulido). However, when unique stimuli from the non-target language are artificially translated to the target language, they lead to interference when compared with existing stimuli in the target language (Otwinowska et al.).

### Morpho-Syntax

Six studies focused on CLI in the domain of morpho-syntax, and examined processing of overlapping and unique syntactic structures across the languages of bi- and multilinguals. Of these, three studies examined CLI in specific syntactic structures in bilingual children (Kubota et al.; Meir and Janssen) and adults (Vingron et al.). Although the studies differ in the tested modalities and in the specific syntactic structures targeted, all three reach similar conclusions in that how and when CLI is evident in the syntactic domain most likely differs across specific linguistic structures (i.e., Language/Item related modulators). Further, all three studies find that the extent of CLI is modulated by individual variability in speakers' profiles of language use and dominance. Vingron et al. also demonstrate such modulating effects in the timing of CLI. Interestingly, in a study extending the investigation of CLI to trilingual speakers, the extent and timing of CLI was also modulated by participants' profile of language dominance (CLI from L1 vs. L2 in L3 processing; Abbas et al.). Finally, Leon Guerrero et al. and Russak and Zaretsky examined CLI in more ecologically valid setting in school aged children (see also Lipner et al., for intervention with school-aged children). They found that meta-linguistic skill, developed in any of a speakers' languages, can holistically support narrative processing in the target language, be it in comprehension or production.

Across the six studies, proficiency emerged as an important modulator of CLI in the morpho-syntactic domain, as exemplified in the direction of CLI from L1 or from L2 (Abbas et al.; Vingron et al.) and as exemplified in individual differences or change over time (Kubota et al.; Meir and Janssen).

## Integrative Summary

Rather than merely documenting the presence of CLI, the current volume explored various factors that might modulate the degree and nature of CLI, namely under what circumstances, for which learners, and in what manner, CLI might be more or less evident. Across 16 independent studies, it becomes clear that there is a high level of interaction between the languages in the multilingual mind, such that the absence of CLI seems to be an exception rather than a norm, although sometimes the observed CLI effects are subtle or weak.

In reviewing the contributions in this volume, we recognize several important common modulators that are linked to the task, the language/item, and the participants of interest. With respect to Task, all studies reviewed here show that whether CLI manifests as facilitation or interference may to a large degree depend on the particular task employed, and especially on what is selected as the baseline against which comparisons are made (see e.g., Du et al. vs. Otwinowska et al.; Hoshino et al.; Iniesta et al.; Woumans et al.). Relatedly, in several cases, dissociations emerged in the patterns of CLI observed as a function of the experimental measure. Manifestations of CLI differed across brain and behavioral indices (Otwinowska et al.), or across offline and more online measures of processing (Abbas et al.).

With respect to Language/Item characteristics, the studies reviewed here convincingly demonstrate CLI across multiple language domains, including lexicon and grammar. However, more work is needed in the domain of phonology as well as in studies including more than a single language domain, to more directly compare the effect of specific modulators across domains. Of note, the studies included in this volume differed in the languages selected as their target (L1/L2/L3), and demonstrate that CLI may take a bidirectional form (Iniesta et al.; Lipner et al.; Whitford and Joanisse; Otwinowska et al.; Vingron et al.). Specifically, dominant L1 influences less dominant L2/L3 processing, but also vice versa, although the latter direction seems weaker (Du et al.). Of note, not all items appear to be affected by CLI to the same extent. This is especially evident in the morpho-syntactic domain, where structure related differences appear to modulate the observed effects (Kubota et al.; Meir and Janssen).

With respect to Participant characteristics, and related to the issue of language direction of CLI noted above, participants' proficiency profile emerges as an important modulator (Abbas et al.; Vingron et al.). Further, language background—namely whether the language was learned as a heritage language or via school-based learning appears to exert an effect (e.g., Fricke). Relatedly, language immersion has been highlighted as crucial for understanding when and how CLI is manifested (Kubota et al.; Meir and Janssen). Indeed, recent work highlights the relevance of patterns of language use as affecting multilingual performance (Gullifer et al., 2018; Beatty-Martínez et al., 2020) and more work is needed to understand their role in possibly modulating CLI. Additionally, we suggest that future work examine whether individual differences in executive control might also modulate the expression of CLI (Prior et al., 2017).

The body of literature included in this volume highlights the contribution of these three types of modulators, and we therefore advocate for including them in future theoretical and empirical work on CLI. Interestingly, two other issues emerge from the integration of the studies in the current volume. First, evidence for CLI observed at a given point in time might in fact reflect processes that had taken place during learning of the relevant linguistic representations, or may reflect concurrent activation across languages as processing unfolds (for further discussion see Du et al.). Moreover, prior linguistic knowledge may exert differential influence depending on the timing at which it occurs (see Marian et al.). Second, whereas the most common approach to examining CLI focuses on linguistic knowledge/representations, the current volume also documents instances where prior linguistic experience exerted its influence via previously practiced skills (Leon Guerrero et al., Lipner et al., Russak and Zaretsky; for discussion see Hirosh and Degani, 2018).

To conclude, the current volume brings together research from diverse perspectives and domains, once again underscoring the critical role of CLI for understanding multilingual processing. The unique contribution of the current volume is in emphasizing that CLI is not a monolithic phenomenon, but rather varies in systematic ways as a function of task, language and participant. These and related modulators should be embraced in future work on multilingual learning and processing. In particular, we suggest that a concentrated effort examining the effect of a selected modulator (e.g., task demands) across different levels of other modulators (Language/Item and Participant characteristics) may be the most fruitful pathway to move the field forward.

## Author Contributions

All authors listed have made a substantial, direct, and intellectual contribution to the work and approved it for publication.

## Funding

ZW was supported by the grant SONATA BIS no. 2015/18/E/HS6/00428 from the National Science Centre Poland. AP and TD were supported by grant 340/18 from the Israel Science Foundation.

## Conflict of Interest

The authors declare that the research was conducted in the absence of any commercial or financial relationships that could be construed as a potential conflict of interest.

## Publisher's Note

All claims expressed in this article are solely those of the authors and do not necessarily represent those of their affiliated organizations, or those of the publisher, the editors and the reviewers. Any product that may be evaluated in this article, or claim that may be made by its manufacturer, is not guaranteed or endorsed by the publisher.
